# Bias field tailored plasmonic nano-electrode for high-power terahertz photonic devices

**DOI:** 10.1038/srep13817

**Published:** 2015-09-08

**Authors:** Kiwon Moon, Il-Min Lee, Jun-Hwan Shin, Eui Su Lee, Namje Kim, Won-Hui Lee, Hyunsung Ko, Sang-Pil Han, Kyung Hyun Park

**Affiliations:** 1 THz Photonics Creative Research Center, Future Research Creative Laboratory, Electronics and Telecommunications Research Institute (ETRI), Daejeon 305-700, Korea

## Abstract

Photoconductive antennas with nano-structured electrodes and which show significantly improved performances have been proposed to satisfy the demand for compact and efficient terahertz (THz) sources. Plasmonic field enhancement was previously considered the dominant mechanism accounting for the improvements in the underlying physics. However, we discovered that the role of plasmonic field enhancement is limited and near-field distribution of bias field should be considered as well. In this paper, we clearly show that the locally enhanced bias field due to the size effect is much more important than the plasmonic enhanced absorption in the nano-structured electrodes for the THz emitters. Consequently, an improved nano-electrode design is presented by tailoring bias field distribution and plasmonic enhancement. Our findings will pave the way for new perspectives in the design and analysis of plasmonic nano-structures for more efficient THz photonic devices.

The terahertz (THz) spectral region has attracted the attention of many researchers in various fields of science and industry because of its merits in accessing low-energy phenomena, uniqueness in material contrast, and ability to penetrate non-polar materials^1^,^2^. The areas of application in the scientific arena include vibration of bio-molecules^3^, carrier dynamics in semiconductor thin-layers^4^, low dimensional materials^5^,^6^, and metal-to-insulator transition in solid-state phase-transition materials^7^,^8^. In industry, THz imaging has been used for quality control^9^,^10^, non-contact thickness measurement^11^,^12^, and biomedical^13^ and security applications^14^. For the various THz applications, the development of intense THz emitters as well as sensitive detectors is very important.

As a result of the efforts of the past decades, a variety of THz devices are available nowadays^1^. Regarding spectral coverage, size, and cost, photoconductive antennas (PCAs)^15^,^16^,^17^ and photomixers (PMs)^18^,^19^,^20^ are the two most distinct categories of THz emitters and detectors. PCAs are used in the generation and detection of broadband THz pulses in THz time-domain spectroscopy (THz-TDS) systems^2^ using femtosecond lasers. PMs generate and detect continuous-wave THz radiation in THz frequency-domain systems (THz-FDS) using an optical beating generated by the superposition of two frequency-tuneable lasers^18^ or a dual-mode laser^19^,^20^. Although there are many structural differences between PCAs and PMs, for example, integration of broadband antennas^21^, both devices mutually rely on the transient movement of photocarriers driven by optical excitations.

Many studies have been conducted on the carrier dynamics in THz PCAs^15^,^16^,^17^, including the effects of carrier lifetime, optical fluence, and bias voltage, both theoretically and experimentally. It is now generally accepted that the photocarriers generated near the metallic electrode are very important for THz generation and detection, which is primarily because of the limited electron velocity and the sub-picosecond carrier lifetime that is required for the THz devices.

As nano-fabrication techniques advance, manipulation of the interactions between the optical excitation and the photoconductive antenna has become possible in many different ways. Recently, various kinds of nano-structures have been applied to THz PCAs^22^,^23^,^24^,^25^,^26^ and PMs^27^,^28^,^29^. The sizes and shapes of the applied nano-structures are diverse, and the enhancements to the conventional devices also range from approximately twice^23^ to more than a factor of 50^24^,^27^. However, nano-structured PCAs and PMs are still in their early stages, and remarkable improvements may yet occur in the future after the underlying physics is experimentally and theoretically clarified.

In nano-structured THz devices, the enhancement in the emission power has been attributed to locally enhanced photocarrier generation through plasmonic field enhancement^23^,^24^,^27^ and the contributions of locally enhanced static bias field around the metallic nano-structure has not been properly considered^27^,^28^. But in real devices, separating those two effects is very difficult because they are mixed by the device geometry, and the carrier dynamics are significantly affected by the operating conditions, such as optical power density and bias^15^,^16^,^17^. Moreover, the antenna geometry, which couples the THz wave to free space, significantly affects the THz emission efficiency^21^,^30^,^31^. In such complex devices, theoretical and numerical study are challenging because the interplay between two significantly different electromagnetic waves, THz wave and infrared, and carrier dynamics in unusual host materials such as low-temperature-grown (LTG) GaAs^32^ and InGaAs^33^ must be solved self-consistently.

In this paper, we show by systematic experimental investigation of three different kinds of nano-electrodes that the role of plasmonic field enhancement in enhancing the power of THz emissions is limited and, should be assisted with bias field enhancement to be effective. We also found out that the effect of local bias field is significant compared to the plasmonic effect under reduced optical power densities. Complemented with three-dimensional numerical studies, the effects of local bias field distribution and plasmon-enhanced local absorption are reasonably clarified, and the effects of the two enhancement mechanisms under various operating conditions are also compared and discussed. We propose a bias field tailored nano-plasmonic electrode structure that exhibits much suppressed saturation under high optical power densities. To maximize the output power by the bias field effect, we propose a large-aperture PCAs adopting the nano-structures to reduce the average optical power density. As a result, the large-aperture nano-PCA delivered a 3-THz bandwidth THz pulse with 240 μW average power by 350 mW optical excitation. This is more than 50 times the power and efficiency of a commercial device of the same aperture size^34^.

## Result

### Design and fabrication of the devices

**Figure 1:**
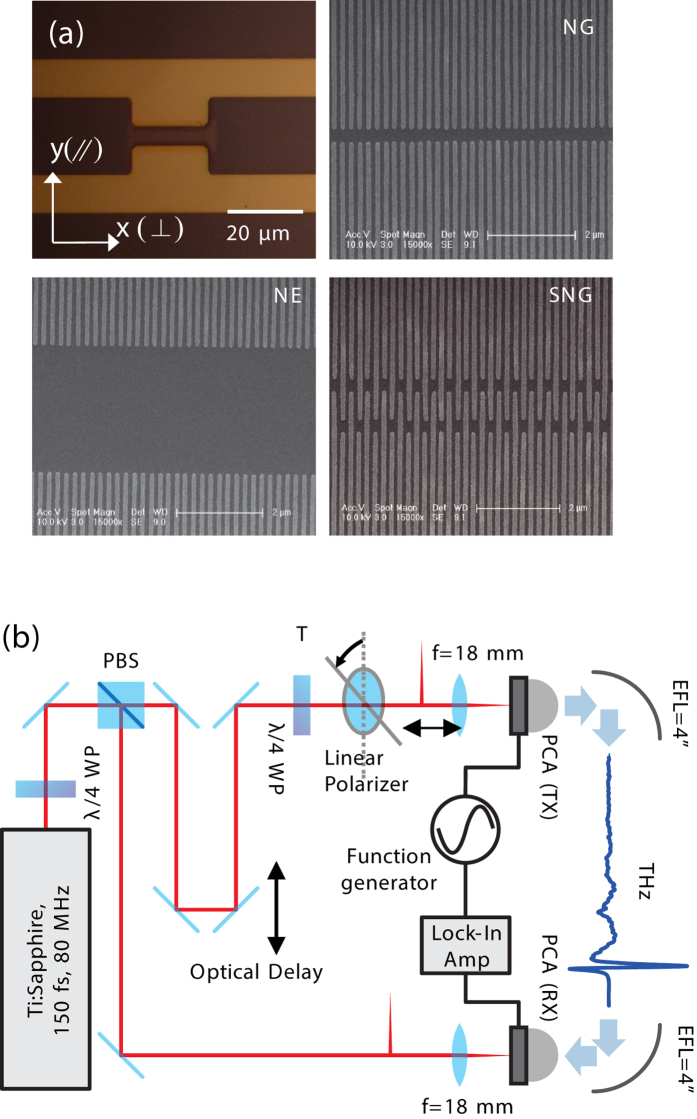
(**a**) Optical microscope images of H-dipole structure and SEM of the fabricated nano-electrodes. (**b**) Schematic of the THz-TDS system used in this study.

We designed three kinds of metallic nano-electrodes and integrated them with the conventional H-dipole shaped THz PCAs. The optical microscope image of the H-dipole structure and the SEM images of each nanostructure are shown in Fig. 1(a), designated by nano-gap (NG), nano-electrode (NE), and shifted nano-gap (SNG) structure. The nano-electrodes are based on nano-fingers of 200 nm periods to maximize the generation of plasmonic photocarriers along both sides of the nano-fingers^23^. The gap between electrodes was set at 200 nm and 3 μm for the NG structure and the NE structure, respectively. In the NE structure, the intermediate region acts as an absorption layer for the ordinary photo-absorption. The SNG structure is designed with a 1-μm wide inter-digit region by alternatively shifting the nano-fingers of the NG structure in the parallel direction to form an inter-digit region. The distribution of plasmonic carriers and bias field then becomes spatially coincident in the inter-digit region to collect the plasmonic carriers more efficiently. A reference PCA of the same geometry was fabricated without nano-fingers. All of the PCAs were fabricated on an Fe-doped semi-insulating GaAs wafer. For THz detection, we fabricated another H-shaped PCA on a low-temperature grown GaAs of sub-picosecond carrier lifetime.

### Measurement of the fabricated PCAs

**Figure 2:**
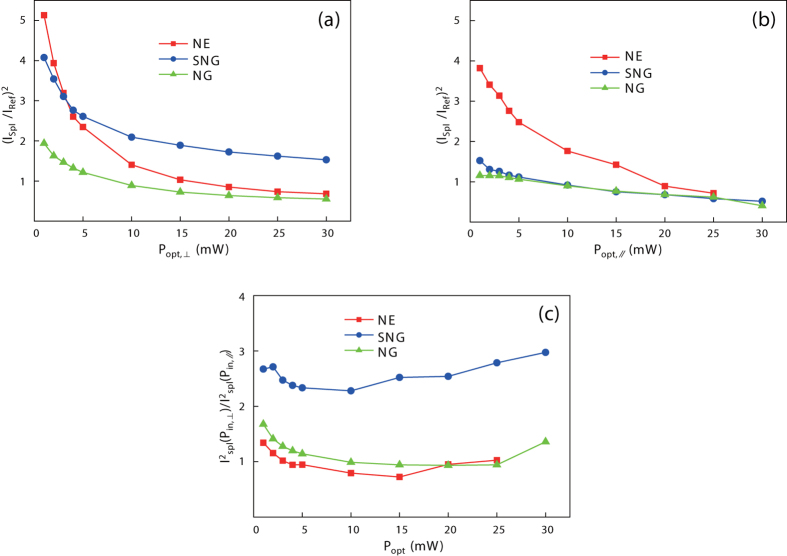
Relative power of the nano-PCAs for optical excitation of (a) perpendicular polarization, and (b) parallel polarization. (**c**) THz power ratio between the perpendicular and the parallel polarization of the optical excitation.

The THz pulses emitting from each PCA were measured using the THz-TDS system depicted in Fig. 1(b). (The details of the experimental setup are discussed in the methods.) To vary the polarization and power of the optical excitation without distorting optical alignment, for experimental accuracy, we inserted a linear polarizer, a quarter-wavelength waveplate, and a variable optical attenuator. In this study, we measured the peak-to-peak THz current (*I*_THz_) as a function of the optical excitation power (*P*_opt_) for the perpendicular and parallel polarizations of which definitions are depicted in Fig. 1(a). The AC bias voltage was set to 8 V_pp_. From these results, we derived the ratios of the THz power to that from the reference PCA for each structure for perpendicular and parallel polarizations, as shown in Fig. 2(a,b). The ratios between these two polarizations are depicted in Fig. 2(c). For the reference measurement, the polarization was set to parallel for better emission efficiency. Time-domain curves and spectra obtained at 10 mW of *P*_opt_ are shown in Fig. S1, and the absolute values of the measured *I*_THz_ are shown in Fig. S2 (see Supporting Information).

When the excitation power is relatively low (*P*_opt_ < 5mW), the enhancement in the THz power via the nano-electrodes is very clearly seen. However, as the optical excitation power increases, saturations in the power enhancement can be observed. The level of saturation depends on the shape of the nanostructure and the polarization of the optical excitation. Irrespective of the polarization, the NE structure was most efficient for *P*_opt_ less than 5 mW. The SNG structure was effective as well, and exhibited less saturation with clear polarization dependence. As a result, the SNG structure was most effective for high optical power with perpendicular polarization. The NG structure proved to be the least effective structure. As clearly shown in Fig. 2(c), in contrast to the others, the SNG structure uniquely exhibits strong polarization dependence.

There are several points to be noted. First, the polarization dependence was only significant for the SNG structure. Second, the NG structure was the least efficient structure, in spite of its strong bias field enhancement due to the narrow gap with large nano-electrode area. Finally, the NE structure is prone to more significant saturation than the other structures. These important observations can be reasonably explained. For this purpose, we numerically calculated the optical field of both polarizations and the bias field distribution in the vicinity of the nano-fingers using a commercial finite element solver (see methods).

### Numerical simulation and discussion

**Figure 3:**
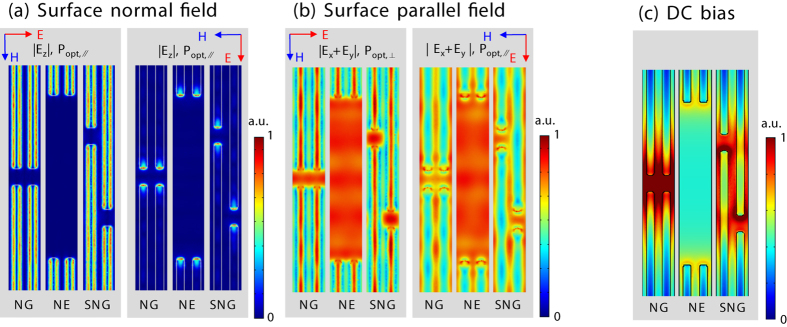
Optical near-field distribution within the GaAs substrate: (**a**) |***E***_z_| distributions for both polarizations. (**b**) |***E***_*x*_ + ***E***_*y*_| distributions for both polarizations. (**c**) Bias field distribution.

The calculated field distributions on the *x*-*y* plane at 10 nm beneath the surface of the GaAs are shown in Fig. 3. It is hard to strictly distinguish the plasmonic field distribution and the ordinary optical field distribution from the numerical simulation results. But by noting that the plasmonic effect induces the strong electric field normal to the metallic surface, we could infer the distribution of plasmonic field, or plasmonic carriers from the |***E***_z_| distribution shown in Fig. 3 (a). The |***E***_x_ + ***E***_y_| distribution, shown in Fig. 3(b), collectively shows the plasmonic field and the normal field distribution near to the nano-fingers. In the most area of absorption region of the NE structure, however, the distribution is not related to the plasmonic effect which is clearly confirmed by the negligible |***E***_z_| within the region. Therefore, |***E***_x_ + ***E***_y_| distribution within the intrinsic region of the NE structure can be asymptotically regarded as the ordinary photo-carrier, or at least ordinary photo carrier-dominant, distribution.

As shown in Fig. 3(a), the |***E***_z_| distribution strongly depend of the polarization of the incident optical field. For the perpendicular polarization, strong |***E***_z_| is induced along both sides of the nano-fingers, indicating the plasmonic enhancement: for the parallel polarization, |***E***_z_| is exclusively induced at the apexes of the nano-fingers, indicating apex-localized plasmonic enhancement. These are well-known phenomena for nano-electrode structures^23^,^24^. Conversely, in the |***E***_*x*_ + ***E***_*y*_| distributions shown in Fig. 3(b), the polarization dependencies are not significant: the absorption regions that are not covered by the metal electrodes are virtually uniformly illuminated. Considering that the color codes in Fig. 3(a,b) are the same, these results can be interpreted as the number of photocarriers generated from the illuminating light may be superior to that from the field enhanced by the plasmons. This illuminating light dominant picture can explain the virtually polarization-insensitive responses in the NE and NG structures in Fig. 2(c). However, for the SNG structure that is highly polarization-dependent, further explanation is necessary.

Consider the bias field distributions in Fig. 3(c). The field enhancements at the tips of the nano-fingers are clearly observed. In the SNG structure, in particular, additional field enhancements along the sides of electrodes within the inter-digit region can be observed. If we assume that the carriers in the bias field enhanced region, irrespective of the involved mechanism, are relevant for THz emission, many of the experimental observations are reasonably explained.

The largest absorption area of the NE structure permits the greatest number of generated photocarriers, which explains the largest power enhancement under low optical excitation densities. In addition, the point-like nature of the apex of the nano-electrodes permits a locally enhanced bias field, which enhances carrier collections under low optical power density. However, as the optical power increases, the bias screening effect^16^ becomes serious because the density of photo-generated carriers and the charge screening increase significantly near the collection point. This explains the earliest saturation of the NE structure. From this, the small absorbing area and localized bias field enhancement makes it clear that the NG structure is the least effective.

By contrast, in the SNG structure, the plasmonic carriers generated on both sides of the nano-fingers, as inferred by Fig. 3(a), can be collected through the apex and the sides of the inter-digit region of the electrodes, as depicted in Fig. 3(c). Because of the increased collection area compared to the NG and NE structures, the SNG structure saturates slowly, as illustrated in Fig. 2(a). Moreover, from the polarization dependence for SNG, we can presume that the integral of the spatial coincidence between the plasmonic and bias field enhancements are very important parameters in the design of the nano-electrode.

From our experiments and numerical simulations, we concluded that both the field enhancement and the local carrier distributions are important for the THz generation. To estimate the maximum achievable enhancement in the terahertz emission power, more rigorous theoretical studies are required. Although the experimental results are roughly understood by the simple assumption, the nano-scale carrier dynamics leave much to be explored. In the NE structure, only a part of the carriers in the absorption region would contribute to the THz emission, owing to the limited electron velocity and the sub-picosecond characteristic time of the THz pulse emission. Thus, the finger period and absorption area of the NE structure should be optimized further. Another important issue is the carrier lifetime. In the THz detectors, a substrate of sub-picosecond carrier lifetime was mandatory. However, recently, a THz detector with a metallic nano-structure^22^ that utilizes no defect-incorporated host material was demonstrated; which shows that the effective carrier lifetime may be determined by the structure itself if the photo-absorption is sufficiently confined around the nano-structures. This is an important point to consider in discussions of the usefulness of a nano-electrode structure.

### Nano-electrode large-aperture PCAs

It is clear that under low excitation power, the NE structure is the most efficient because of the locally enhanced bias field rather than the plasmonic effect, while the SNG structure may be more efficient at high optical excitation power owing to the plasmonic effect complemented by the bias field.

**Figure 4:**
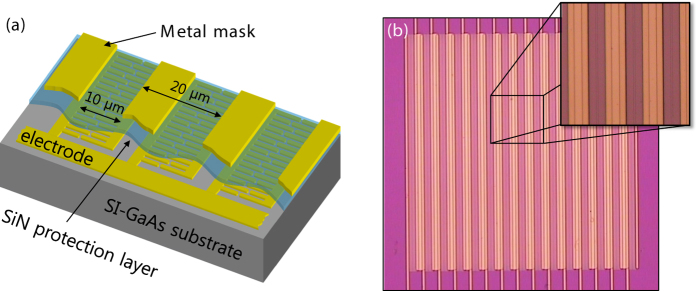
(**a**) Structure of the large-aperture PCA adopting SNG nano-electrode. (**b**) Optical microscope image of the fabricated device.

**Figure 5:**
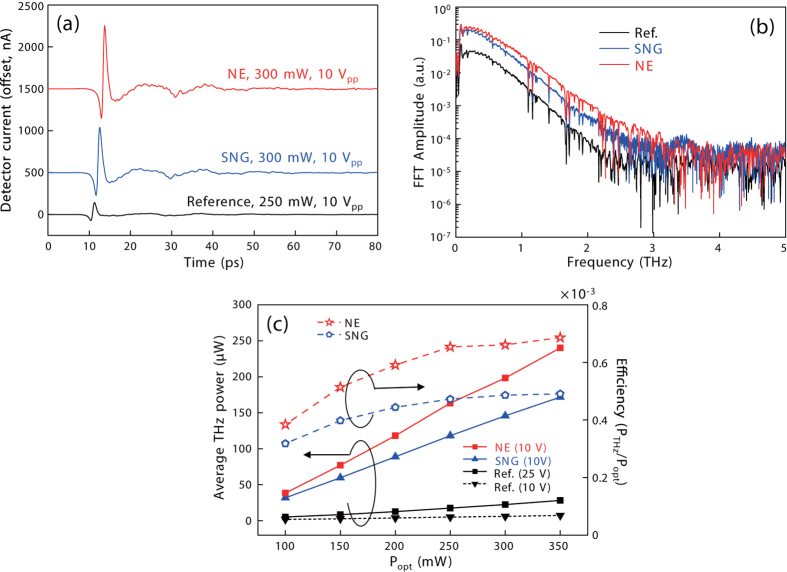
THz emission from the large-aperture PCAs: (**a**) Time-domain curves. (**b**) FFT spectra. (**c**) Absolute power and optical-to-THz conversion efficiency measured as a function of the optical excitation power.

At one extent of these two possibilities, we fabricated nano-electrodes on large-aperture emitters to experimentally confirm the expected enhancement in the low optical power density. For the comparison, we also fabricated a reference large-aperture PCA (L-PCA) without nano-electrodes. The active areas of all the L-PCAs were set to 300 × 300 μm^2^, identical to the size of a commercial large-aperture emitter^34^, which approximately reduces the optical power density to 1/280. The structure and optical microscope image of the SNG-based large-aperture emitter are shown in Fig. 4(a,b), respectively. (Detailed fabrication procedures are given in the methods.) We measured the time-domain curves from the large-aperture emitters, then replaced the receiver with a calibrated commercial pyro-electric detector (THz5I-BL-BNC, Gentec-EO) for absolute power measurements. The results obtained are illustrated in Fig. 5(a–c).

As expected, both PCAs exhibited significantly improved THz emission power compared to the reference. The NE structure was more effective than the SNG structure for the large-aperture emitter, in congruence with our study. At 300 mW optical excitation, *I*_THz_ of the NE PCA was approximately 1200 nA, which is close to the detection limit of our lock-in amplifier, as shown in Fig. 5(a). As shown in Fig. 5(b), the bandwidth was enhanced by 1 THz compared to the reference, reaching up to 3 THz. The dynamic power range was above 70 dB. The measured power is shown in Fig. 5(c). At 350 mW of optical excitation power with AC bias of 20 V_pp_, which corresponds to 10 V of DC bias, the measured average THz powers were 170 μW and 240 μW for the SNG and NE structures, respectively. With the reference PCA, the output powers were 7.3 μW and 28.3 μW at DC bias of 10 V and 25 V, respectively. The polarization of the optical excitation was perpendicular and parallel to the nano-finger direction for the nano-PCAs and the reference PCA, respectively, which are the optimum polarization for each device. Up to the 350 mW of optical excitation, which is the maximum power currently available to us, the THz output power increased linearly without saturation. In the current operating condition, the bias field enhancement is the major enhancement mechanism. However, we expect that the SNG structure may be more effective if the optical excitation is increased further. In fact, the optical-to-THz conversion efficiencies of both nano-structures gradually increased as the optical power increased. Compared to a commercial micro-lens integrated large-aperture PCA (iPCAp-21-05-300-800-h, BATOP GmbH)^34^, the emission bandwidth was marginally better, and the power and efficiency were more than 50 times greater under less bias voltage but with the same optical excitation power. Within the maximum laser power available to us, neither power saturation nor efficiency reduction was observed. Thus, the demonstrated output power was far less than the maximum achievable power.

In addition, we measured the output power as a function of the DC bias (see Supporting Information). The SNG PCA exhibited strong power saturation as the bias increased, whereas the others did not. Below 5 V of DC bias, the output power from the SNG was similar to that of the NE structure. We also measured time-domain traces and spectra as a function of bias voltage and excitation power (see Supporting Information).

## Summary

In this paper, we examined the importance of bias field distributions in power enhancement via plasmonic nano-electrodes for THz PCAs. The contributions of static and plasmonic mechanisms may depend on the operating conditions, such as optical power density and bias level. On the basis of our analysis, we designed and fabricated less-saturating plasmonic electrodes and a large-aperture THz PCA that emits an intense broadband THz pulse. Note that the large-aperture approach can be combined with much higher optical excitation sources of μJ or mJ of pulse energy by simply increasing the aperture area. Our results can be adopted for various kinds of optoelectronic devices, such as light harvesting devices and high-speed photodetectors, as well as THz devices including PMs and PCAs.

The nano-electrode large-aperture PCA may be viewed as an array of infinitesimal dipole antennas of which design parameters and structures can be varied over the aperture area. This leaves diverse room for integrating various functional structures of larger characteristic sizes, including periodic structures such as photonic crystals^35^,^36^ and meta-materials^37^,^38^. In addition, it may be possible to further tailor THz radiation characteristics including emission power, phase, and spectral bandwidth.

## Methods

### Device Fabrication

We fabricated the nano-electrodes on an Fe-doped semi-insulating GaAs (SI-GaAs) wafer by electron-beam lithography using PMMA and subsequent e-beam evaporation of a 25-nm gold layer with a 5-nm chrome inter-layer for efficient adhesion. The resistivity and mobility of the SI-GaAs wafer were greater than 10^8^ Ω-cm and 5500 cm^2^V^−1^s^−1^, respectively. Then, the H-dipole structure was patterned with a contact aligner using an image-reversal process. Next, a 5-nm chrome layer and a 300-nm gold layer were deposited, followed by a lift-off process. The gap between the electrodes of the H-dipole PCA was 5 μm. For comparison, we also fabricated the same PCA structure on another SI-GaAs wafer, without fabricating any kind of nano-electrode. For the THz pulse emission, we confirmed that the pulse from the SI-GaAs based PCA was similar to that of the LTG-GaAs based PCA in terms of peak pulse intensity and emission bandwidth.

The large-aperture PCAs were fabricated in the same manner, with the exception that a 200-nm thick SiN layer was deposited following fabrication of the nano-electrodes. The SiN layer provided a protection layer for the nano-electrodes during the rest of the process. Next, cathode and anode electrodes were patterned on the surface with contact lithography via an image-reversal process. The SiN layer under the electrode pattern was removed by dry etching. A gold layer was then deposited using a 5-nm chrome layer as adhesion promotion layer, followed by a lift-off process. Following formation of the electrode, a 300-nm thick SiN layer was deposited, and the metal mask was patterned to prevent unwanted reverse current. Finally, the SiN on the electrode was removed in order to allow contact. The thickness of the SiN on the nano-electrode was 500 nm in total, which roughly corresponds to the anti-reflection condition at 790 nm. The reference PCA was fabricated in the same manner except for the nano-electrode. For THz detection, we fabricated detector PCAs on an LTG GaAs wafer with a sub-0.5 ps carrier lifetime.

### Measurement of the THz-PCAs

We implemented a conventional THz-TDS system using a Ti:sapphire femtosecond laser (MIRA 900, Coherent, Inc.). The centre wavelength, duration and repetition rate of the laser were 790 nm, 200 fs, and 80 MHz, respectively. Thus, the pulse energy was about 4.4 nJ at 350 mW average power. Two hyper-hemispheric lenses were used for efficient emission and detection of THz pulses. Two off-axis parabolic mirrors, each with effective focal length 4 inches were used. The average optical power incident on the receiving THz PCA was 12 mW. The average power and polarization of the optical excitation were controlled by inserting a linear polarizer, a variable optical attenuator, and a quarter-wavelength waveplate. Thus, polarization and power to the THz emitter were controlled without altering its optical alignment. For lock-in detection, electric bias to the transmitting PCA was sinusoidally modulated by a function generator at 2 kHz.

Two lenses with focal length 18 mm were used to focus optical excitation, and the diameter of the focused beam was calculated to be 15 μm. For the measuring of the H-dipole PCAs, the laser beam was focused to the gap of 5 μm. Further, for measuring of the large-area PCAs, the focusing lens was retracted by 4 mm to increase the beam diameter to 250 μm. Consequently, the optical power density was reduced by a factor of 280, compared to the tight-focusing condition. In practice, the laser beam scattered by the PCA structure was projected on a white screen, which had a through-hole for the laser to pass. The beam size was confirmed via Gaussian-optic calculations.

A commercial pyro-electric detector (THz5I-BL-BNC, Gentec-EO) was used to measure the THz power. A calibration datasheet provided by the detector’s vendor stated that its sensitivity was 54.5 kV/W at 632 nm with response time less than 0.2 s. At 30 THz, absorbance of the detector is more than 90%; however, in the region between 0.1–1 THz, the absorption becomes less than 90%. It is possible that the actual output power would be better than that presented in Fig. 5(c). For the measurement, the electric bias was modulated at 5 Hz and an oscilloscope was used to measure the output voltage of the pyro-electric detector.

### Numerical Simulation

To simulate the three-dimensional device for calculation of the optical field enhancement around the nano-electrodes and bias field distributions, we used several approximations and simplifications in order to reduce the computational burdens. Throughout the simulations, the thin (approximately 5 nm) adhesive chromium layer between the gold electrode and semiconductor substrate was ignored in order to reduce the computational complexity. In addition, the distance between the base-electrodes was reduced from the actual distance of 10 μm to 4 μm in the simulations. The simulations were conducted using finite element method-based commercial software (Comsol Multiphysics, www.comsol.com). To model periodic structures, we used Bloch periodic boundary conditions to the faces normal to the x-axis (in Fig. 1(a)). Even though the periods for NG and NE are 2*w*, where *w* = 100 nm is the width of nanoelectrode, for the fair comparisons of different electrode patterns including SNG with reduced the numerical errors, the periods for all the structures of NG, NE, and SNG are taken as 4 *w*. For the simulation, the incident field was assumed as a continuous wave of which center wavelength is 790 nm. At this wavelength, the values for the relative electric permittivities for the gold and GaAs are chosen from literatures as -26.2760-1.8496*i* and 13.5350-0.6781*i*, respectively^39^,^40^. The negative signs in the imaginary parts of the permittivity values are used to express the lossy materials under the convention of Comsol. For the simulations of DC responses, we imposed 1V potential difference between upper (y-positive sides) and lower (y-negative sides) electrodes.

## Additional Information

**How to cite this article**: Moon, K. *et al.* Bias field tailored plasmonic nano-electrode for high-power terahertz photonic devices. *Sci. Rep.* **5**, 13817; doi: 10.1038/srep13817 (2015).

## Supplementary Material

Supplementary Information

## References

[b1] Tonouchi M. Cutting-edge terahertz technology. Nat. Photon 1, 97–105 (2007).

[b2] Ferguson B. & Zhang X.-C. Materials for terahertz science and technology. Nat. Mater 1, 26–33 (2002). 12618844 10.1038/nmat708

[b3] Walther M., Plochocka P., Fischer B., Helm H. & Jepsen P. U. Collective vibrational modes in biological molecules investigated by terahertz time-domain spectroscopy. Biopolymers 67, 310–313 (2002). 12012455 10.1002/bip.10106

[b4] Ulbricht R., Hendry E., Shan J., Heinz T. F. & Bonn M. Carrier dynamics in semiconductors studied with time-resolved terahertz spectroscopy. Rev. Mod. Phys. 83, 543–586 (2011).

[b5] Lloyd-Hughes J. Terahertz spectroscopy of quantum 2D electron systems. J. Phys. D: Appl. Phys. 47, 374006 (2014).

[b6] Maeng I. *et al.* Gate-controlled nonlinear conductivity of Dirac fermion in graphene field-effect transistors measured by terahertz time-domain spectroscopy. Nano Lett. 12, 551–555 (2011). 22214292 10.1021/nl202442b

[b7] Jepsen P. U. Metal-insulator phase transition in a VO2 thin film observed with terahertz spectroscopy. Phys. Rev. B 74, 205103 (2006).

[b8] Mandal P., Speck A., Ko C. & Ramanathan S. Terahertz spectroscopy studies on epitaxial vanadium dioxide thin films across the metal-insulator transition. Opt. Lett. 36, 1927–1929 (2011). 21593938 10.1364/OL.36.001927

[b9] Hu B. B. & Nuss M. C. Imaging with terahertz waves. Opt. Lett. 20, 1716–1718 (1995). 19862134 10.1364/ol.20.001716

[b10] Han S.-P. *et al.* Real-time continuous-wave terahertz line scanner based on 1×240 InGaAs Schottky barrier diode array detector. Opt. Express 22, 28977–28983 (2014). 25402136 10.1364/OE.22.028977

[b11] Moon K. *et al.* Continuous-wave terahertz system based on a dual-mode laser for real-time non-contact measurement of thickness and conductivity. Opt. Express 22, 2259–2266 (2014). 24663518 10.1364/OE.22.002259

[b12] Lee I.-M. *et al.* Frequency modulation based continuous-wave terahertz homodyne system. Opt. Express 23, 846–858 (2015). 25835845 10.1364/OE.23.000846

[b13] Pickwell E. & Wallace V. P. Biomedical applications of terahertz technology. J. Phys. D: Appl. Phys. 39, R301–R310 (2006).

[b14] Federici J. F. *et al.* THz imaging and sensing for security applications—explosives, weapons and drugs. Semicond. Sci. Technol. 20, S266–S280 (2005).

[b15] Rodriguez G., Caceres S. R. & Taylor A. J. Modeling of terahertz radiation from biased photoconductors: transient velocity effects. Opt. Lett. 19, 1994–1996 (1994). 19855720 10.1364/ol.19.001994

[b16] Rodriguez G. & Taylor A. J. Screening of the bias field in terahertz generation from photoconductors. Opt. Lett. 21, 1046–1048 (1996). 19876247 10.1364/ol.21.001046

[b17] Jepsen P. U., Jacobsen R. H. & Keiding S. R. Generation and detection of terahertz pulses from biased semiconductor antennas. J. Opt. Soc. Am. B 13, 2424–2436 (1996).

[b18] Brown E. R., Mclntosh K. A., Nichols K. B. & Dennis C. L. Photomixing up to 3.8 THz in low-temperature-grown GaAs. Appl. Phys. Lett. 66, 285 (1995).

[b19] Kim N. *et al.* Tunable continuous-wave terahertz generation/detection with compact 1.55 μm detuned dual-mode laser diode and InGaAs based photomixer. Opt. Express 19, 15397–15403 (2011). 21934903 10.1364/OE.19.015397

[b20] Moon K. *et al.* Low-temperature-grown InGaAs terahertz photomixer embedded in InP thermal spreading layer regrown by metalorganic chemical vapor deposition. Opt. Lett. 38, 5466–5469 (2013). 24343018 10.1364/OL.38.005466

[b21] Gregory I. S. *et al.* Optimization of photomixers and antennas for continuous-wave terahertz emission. IEEE J. Quantum Electron 41, 717–728 (2005).

[b22] Heshmat B. *et al.* Nanoplasmonic terahertz photoconductive switch on GaAs. Nano Lett. 12, 6255–6259 (2012). 23171276 10.1021/nl303314a

[b23] Park S.-G. *et al.* Enhancement of terahertz pulse emission by optical nanoantenna. ACS Nano 6, 2026–2031 (2012). 22339093 10.1021/nn204542x

[b24] Berry C. W. *et al.* Significant performance enhancement in photoconductive terahertz optoelectronics by incorporating plasmonic contact electrodes. Nat. Comm. 4, 1622, 10.1038/ncomms2638 (2013). 23535643

[b25] Yang S.–H., Hashemi M. R., Berry C. W. & Jarrahi M. 7.5% optical-to-terahertz conversion efficiency offered by photoconductive emitters with three-dimensional plasmonic contact electrodes. IEEE Trans. Terahertz Sci. Technol. 4, 575–581 (2014).

[b26] Jooshesh A. *et al.* Nanoplasmonics enhanced terahertz sources. Opt. Express 22, 27992 (2014). 25402040 10.1364/OE.22.027992

[b27] Tanoto H. *et al.* Nano-antenna in a photoconductive photomixer for highly efficient continuous wave terahertz emission. Sci. Rep. 3, 2824, 10.1038/srep02824 (2013). 24100840 PMC3792413

[b28] Khiabani N. *et al.* A novel sub-THz photomixer with nano-trapezoidal electrodes. IEEE Trans. Terahertz Sci.Technol. 4, 501–508 (2014).

[b29] Berry C. W. *et al.* Plasmonics enhanced photomixing for generating quasi-continuous-wave frequency-tunable terahertz radiation. Opt. Lett. 39, 4522–4524 (2014). 25078218 10.1364/OL.39.004522

[b30] Pastol Y., Arjavalingam G. & Halbout J.–M. Characterization of an optoelectronically pulsed equiangular spiral antenna. Electron Lett. 26, 133–135 (1990).

[b31] Dykaar D. R. *et al.* Log-periodic antennas for pulsed terahertz radiation. Appl. Phys. Lett. 59, 262–264 (1991).

[b32] Melloch M. R. *et al.* Low-temperature grown III-V materials. Annu. Rev. Mater. Sci. 25, 547–600 (1995).

[b33] Metzger R. A., Brown A. S., McCray L.G. & Henige J.A. Structural and electrical properties of low temperature GaInAs. J. Vac. Sci. Technol. B 11, 798–801 (1993).

[b34] Matthäus G. *et al.* Microlens coupled interdigital photoconductive switch. Appl. Phys. Lett. 93, 091110 (2008).

[b35] Chassagneux Y. *et al.* Electrically pumped photonic-crystal terahertz lasers controlled by boundary conditions. Nature 457, 174–178 (2009). 19129844 10.1038/nature07636

[b36] Kakimi R., Fujita M., Nagai M., Ashida M. & Nagatsuma T. Capture of a terahertz wave in a photonic-crystal slab. Nat. Photon 20, 657–663 (2014).

[b37] Chen H.-T. *et al.* Active terahertz metamaterial devices. Nature 444, 597–600 (2006). 17136089 10.1038/nature05343

[b38] Withayachumnankul W. & Abbott D. Metamaterials in the terahertz regime. IEEE Photonics J 1, 99–118 (2009).

[b39] Palik E. D. in Handbook of Optical Constants of Solids 286–287 (Academic Press, 1991).

[b40] Adachi S. in Optical Constants of Crystalline and Amorphous Semiconductors Vol. 1 220–226 (Kluwer Academic Publishers, 1999).

