# Supplementary material for: Bias field tailored plasmonic nano-electrode for high-power terahertz photonic devices
Source: Sci Rep. 2015 Sep 8;5:13817. doi: 10.1038/srep13817 (PMC4561887; doi:10.1038/srep13817)
Supplement: Supplementary Information [file srep13817-s1.pdf]

Supplementary Information

Bias field tailored plasmonic nano-electrode for high-power terahertz photonic devices

Kiwon Moon, Il-Min Lee, Jun-Hwan Shin, Eui Su Lee, Namje Kim, Won-Hui Lee, Hyunsung Ko, Sang-Pil Han, and Kyung Hyun Park[*]

THz Photonics Creative Research Center, Future Research Creative Laboratory, Electronics and Telecommunications Research Institute (ETRI), Daejeon 305-700, KOREA

*Correspondence to khp@etri.re.kr

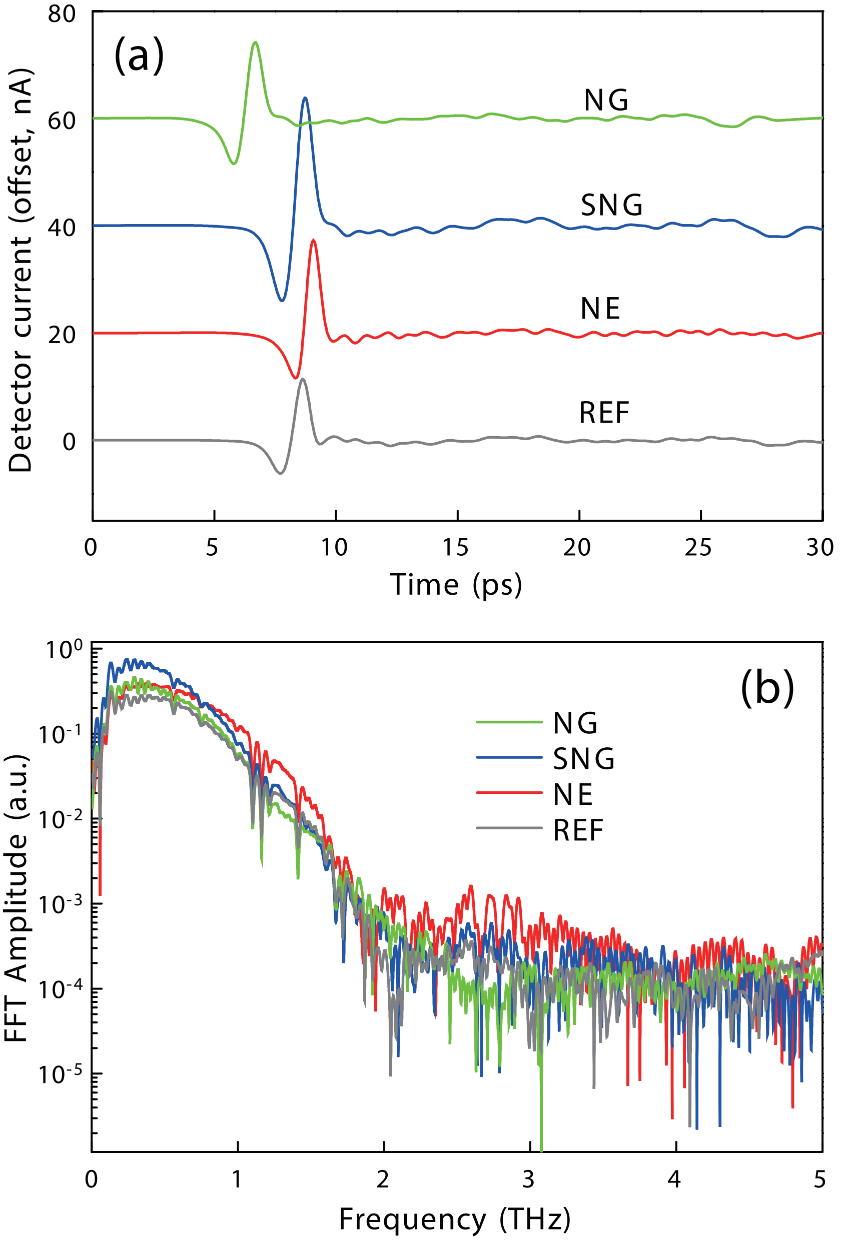

Figure S1. THz-TDS result of the nano-PCAs. The AC electric bias was 8 V_{pp} with 10 mW optical excitation: (a) Time-domain curves. (b) FFT spectra.

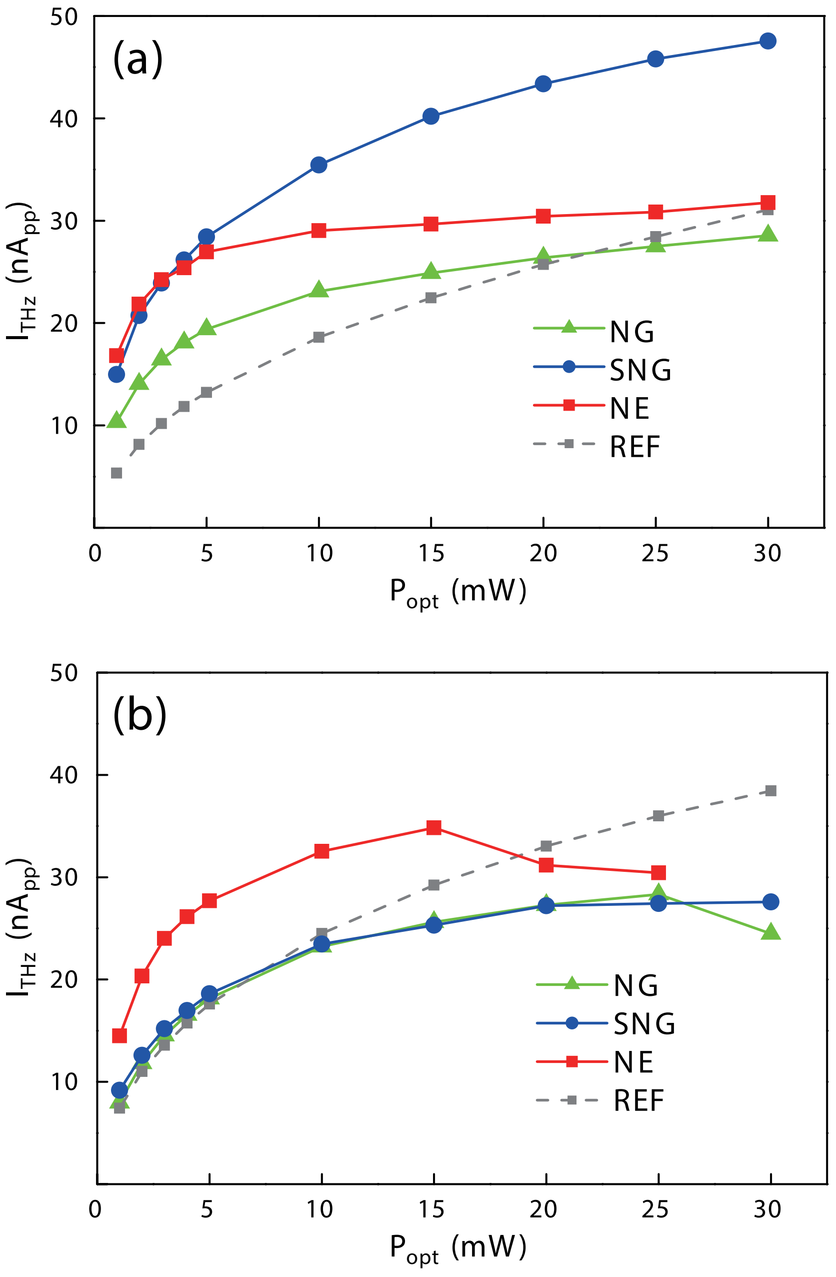

Figure S2. Peak-to-peak current of the THz pulse from each structure, presented as a function of the optical excitation power (a) for the perpendicular polarization and (b) for the parallel polarization.

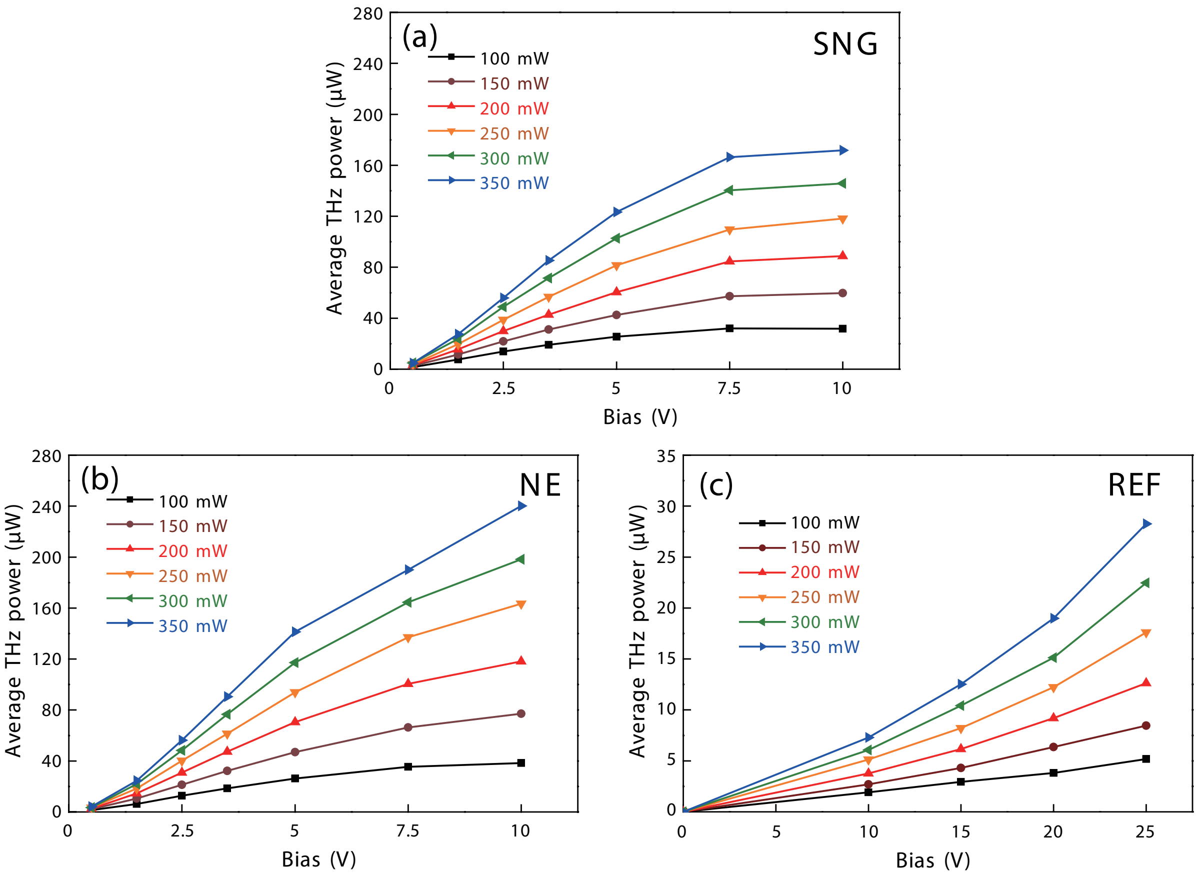

Figure S3. THz output power from large-aperture PCAs measured as a function of the electric bias and the optical excitation power: (a) SNG structure. (b) NE structure. (c) Reference. Note that the NE structure becomes efficient under high electric bias, whereas the SNG structure saturates with respect to the electric bias.

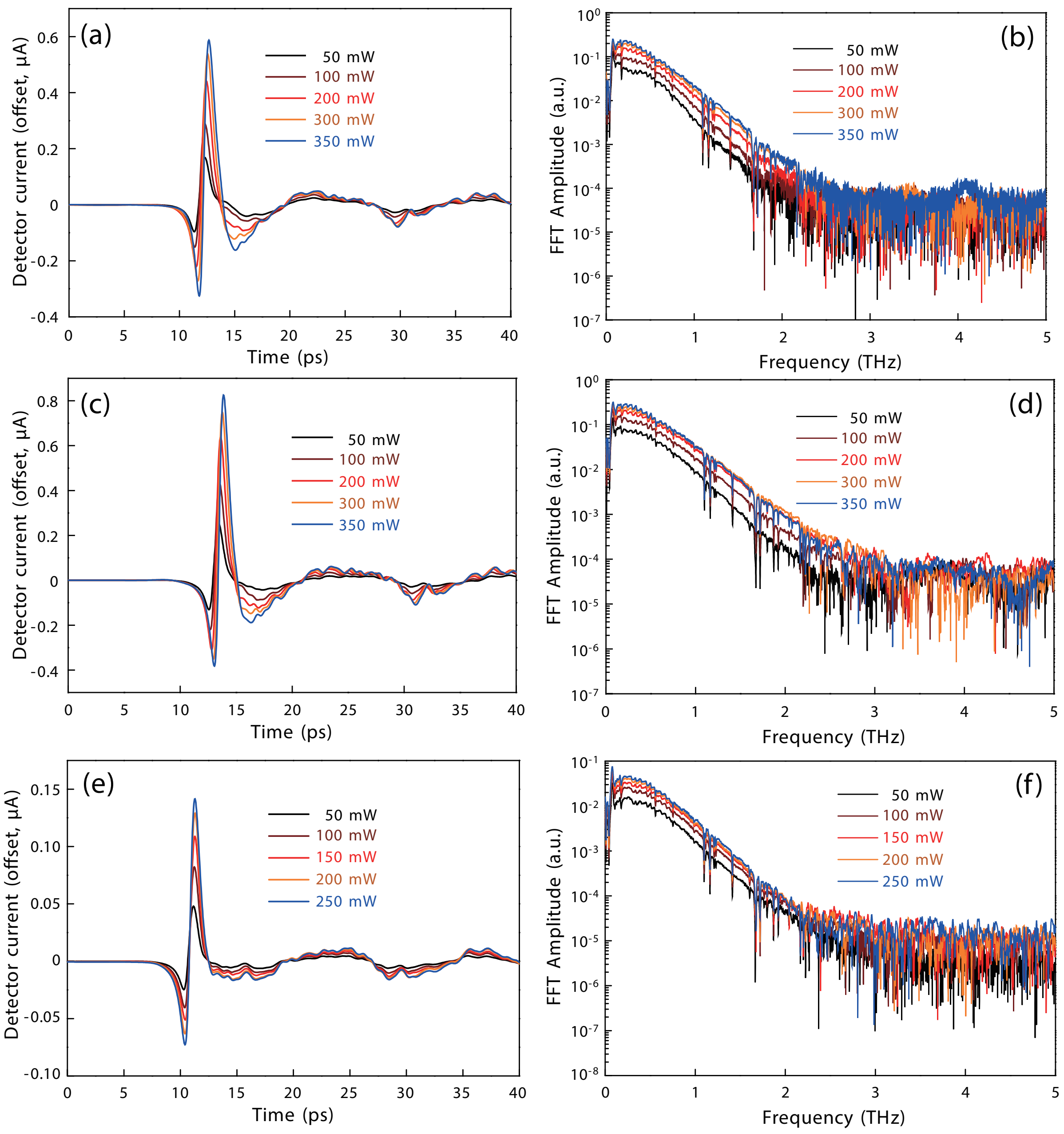

Figure S4. Time-domain curves and FFT spectra, measured as a function of the optical excitation. The AC electric bias was 10 V_{pp}: (a), (b) SNG structure. (c), (d) NE structure. (e), (f) Reference.
